# Duplex Collecting System with Ectopic Ureters Opening into Vagina: A Case Report

**DOI:** 10.31729/jnma.6570

**Published:** 2022-02-28

**Authors:** Suraj Singh, Sulochana Dahal, Anuj Kayastha, Bijay Thapa, Anupama Thapa

**Affiliations:** 1 Department of Surgery, Kanti Children's Hospital, Maharajgunj, Kathmandu, Nepal

**Keywords:** *ectopic*, *ureter*, *urinary incontinence*, *vagina*

## Abstract

Continuous urinary leakage, along with normal deliberate voiding, must suggest diagnosis of ectopic ureter, specifically in girls. Combination of a duplicated collecting system with distal, infra-sphincteric, vaginal insertion of ureter is an uncommon congenital anomaly and rare cause of urinary incontinence. We present a case report of a 7-year-old girl who presented to the urology department with urinary incontinence despite successful toilet training and history of recurrent urinary tract infections. Right duplex collecting system was seen on ultrasound. Magnetic resonance urography revealed a near complete right duplex collecting system with ectopic insertion of ureter into vagina, and aplastic uterus with bilateral normal ovaries suggestive of Mayer-Rokitansky-Kuster-Hauser syndrome. Surgical treatment consisted of "common sheath" reimplantation of ectopic ureters into bladder, with complete resolution of symptoms. This case suggests to us that congenital abnormalities of the genitourinary tract should be considered in case of urinary incontinence and recurrent urinary tract infections.

## INTRODUCTION

The ectopic ureter usually terminates distal to bladder neck in girls, hence it is associated with incontinence as ureteral opening is distal to the sphincter.^1^ In girls 80% of the cases with ectopic ureters are associated with duplex collecting system.^2,3^ Normal urination together with continuous incontinence is pathognomonic feature of infra-sphincteric ureteral openings.^4^ The diagnosis is made usually during childhood because of recurrent urinary tract infections (UTIs) or urinary incontinence.^3^ Magnetic resonance urography has been found to be more useful in depicting ectopic ureter, in detection of subtle duplication of collecting system and in providing a global view of urinary tract anomalies.^5^

## CASE REPORT

A 7 year old female was brought to the urology department of Kanti Children's Hospital by her parents with complaints of continuous low volume urine leakage causing persistent wetting of her underpants. There was no history of urge or stress incontinence. Despite having incontinence, she had normal bladder habits. On examination, the patient's vitals were stable, general condition was fair and without pallor, icterus, cyanosis, dehydration or edema. Systemic examination findings were satisfactory. Also, there was normal external genitalia with no vaginal pooling or any visible ectopic ureteral orifice with intermittent incontinence on increased abdominal pressure.

**Figure 1:**
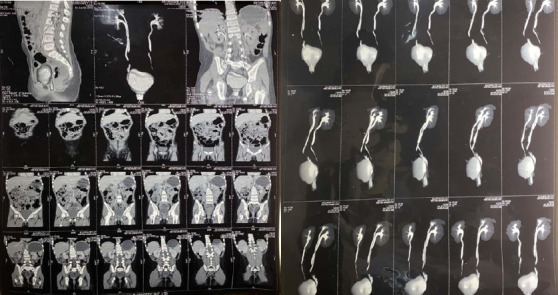
Preoperative CT urogram.

Her parents gave a past history of recurrent febrile urinary tract infections since her infancy. She was under prophylactic antibiotics for urinary tract infection and was prescribed anticholinergics suspecting overactive bladder but the symptoms didn't subside. Here at our hospital, we first advised abdominal ultrasound which revealed the right duplex collecting system. CT urogram was planned which revealed a partial duplex system of the right kidney with ectopic insertion of ureter in the cervix (Figure 1).

**Figure 2:**
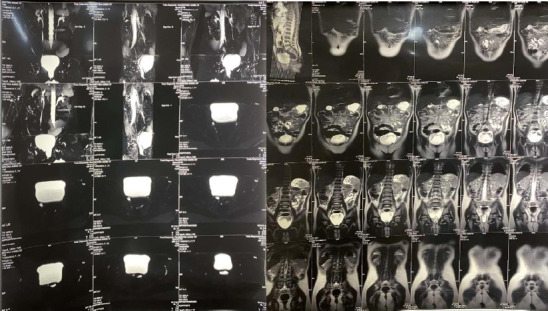
Preoperative Magnetic resonance urography.

To confirm origin, course and insertion of ureters, magnetic resonance urography was done which suggested near complete right duplex collecting system with ectopic insertion of the ureters into lower vaginal canal and aplastic uterus with bilateral normal ovaries suggestive of Mayer-Rokitansky-Kuster-Hauser syndrome (Figure 2).

**Figure 3:**
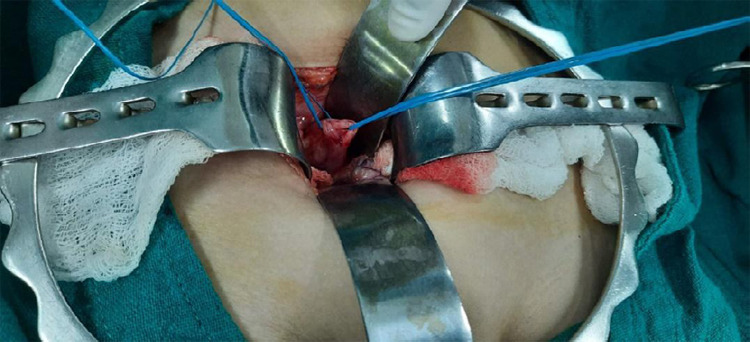
Intraoperative findings revealing duplex ureter.

The child was admitted in a surgical ward and planned for common sheath right ureteral reimplantation in the bladder. Under general anesthesia, Rutherford Morison incision was given, abdomen opened in layers, with intraoperative findings of double ureter (Figure 3) and opening ectopically into vagina which was confirmed by intraoperative instillation of methylene blue dye once at a time through each ureter, dye from both the ureter was visualized coming through vagina with no dye in the urine bag draining urine. Common sheath extra vesical/Lich Gregoir Ureteral Reimplantation was done over a 5Fr Double J stent. The uterus and fallopian tube could not be appreciated. Abdomen was closed in layers and post-operative antibiotics and analgesics were given. The patient was discharged on the 4th postoperative day with no features of incontinence and chemoprophylaxis for one month. On follow up, there was no urinary incontinence and double J stent was removed on the 6^th^ postoperative week.

## DISCUSSION

Duplication of the ureters is a relatively common congenital anomaly but an ectopic ureter, however, is a rare ureteral entity with an estimated incidence of 0.05-0.025%.^6,7^ A duplex collecting system with ectopic ureter opening in the vagina is an extremely rare congenital genitourinary tract abnormality and this is the first such case reported from the country.

In a case report by Duici C, et al. duplex left kidney with double ureters, both inserting together into the vagina was reported.^8^ In our case as well, double ureters originating from a near complete right duplex system inserted into the lower vaginal canal. In their case "en bloc" ureteral reimplantation using the open "Cohen ureteroneocystostomy" was performed. However in our case "common sheath" reimplantation of the ectopic ureters into the bladder using the open "Lich Gregoir ureteroneocystostomy" was performed.

The sites of insertion of ectopic ureters are the bladder neck/urethra (35%), vestibule (30%), vagina (25%) or uterus (5%) in females, and the bladder neck and prostatic urethra (48%), ejaculatory ducts (8%), and vas deferens (3%) in males. Urinary incontinence is the main symptom of an ectopic ureter, especially in females, while in both sexes, an ectopic ureter may present as a prenatal diagnosis because of a urinary tract infection (UTI), or congenital obstructive uropathy which can lead to chronic kidney failure.^2^

Occasionally, although the ureter opens into infra-sphincteric region, incontinence does not manifest itself if it drains an excessively atrophic renal segment or in the presence of compression of lower third of the ureteral segment between muscles of the external sphincter till advanced age. However, they become symptomatic at a later stage in conditions like childbirth which weakens the external sphincter.^9^

The patient's presenting symptoms depend on the insertion site of the ectopic ureter, and this differs between girls and boys.^10^ Usually males do not present with urinary incontinence because of the ureter's insertion above the external urethral sphincter. Similar to our patient, most girls present with urinary dribbling, as the insertion of the ectopic ureter is distal to the urethral sphincter. Mostly the affected girls accompany normal voiding patterns with small volume or spotting incontinence.

Imaging studies are mandatory to confirm the diagnosis. Renal ultrasound represents the initial diagnostic investigation but because of its limitation, it is not helpful. So, contrast-enhanced computed tomography (CT) or magnetic resonance urography should be the method of choice for depicting or ruling out an ectopic ureter.^3-5^

The best treatment in children and symptomatic patients is surgery, and it tries to resolve the incontinence, prevent further complications, preserve renal function and eliminate recurrent UTIs. The surgical management of ectopic ureter depends on surgeon experience and preference, laparoscopic experience, pediatric material investments.^8^

Normally, the duplex system is associated with Weigert Meyer Principle, in which the upper moiety ureter inserts inferomedially and is ectopic while the lower moiety ureter is inserted superolaterally in orthotopic location. In our case, there is near complete duplication of ureters and the common ureter is inserted into the vagina, which does not follow the usual Weigert Meyer Law of Duplex Systems. Our case is not the first case report that does not follow the Weigert-Meyer law. Rarely reported in literature, "ectopic pathway" of Stephens postulates that an ectopic ureter may drain not only distal to the normal ureteric orifice (Weigert - Meyer law) but may also drain medially and superiorly to it (violating Weigert-Meyer law).^8^ Thus, proper medical history and complete clinical evaluation is mandatory.

## References

[1] Berrocal T, Lopez-Pereira P, Arjonilla A, Gutierrez J (2002). Anomalies of the distal ureter, bladder, and urethra in children: embryologic, radiologic, and pathologic features.. Radiographics..

[2] Baskin Laurence S (2016). Ectopic Ureter [Internet]..

[3] Avni EF, Matos C, Rypens F, Schulman CC (1997). Ectopic vaginal insertion of an upper pole ureter: demonstration by special sequences of magnetic resonance imaging.. J Urol..

[4] Kibar Y, Avci A, Akay O, Dayang M (2005). Dribbling of urine due to ectopic vaginal insertion of an upper pole ureter diagnosed by magnetic resonance urography.. Int Urol Nephrol..

[5] Avni FE, Nicaise N, Hall M, Janssens F, Collier F, Matos C (2001). The role of MR imaging for the assessment of complicated duplex kidneys in children: preliminary report.. Pediatr Radiol..

[6] Gay SB, Armistead JP, Weber ME, Williamson BR (1991). Left infrarenal region: anatomic variants, pathologic conditions, and diagnostic pitfalls.. Radiographics..

[7] Mandell J, Bauer SB, Colodny AH, Lebowitz RL, Retik AB (1981). Ureteral ectopia in infants and children.. J Urol..

[8] Duicu C, Kiss E, Simu I, Aldea C (2018). A Rare Case of Double-System With Ectopic Ureteral Openings Into Vagina.. Front Pediatr.

[9] Jain KA (2007). Ectopic vaginal insertion of an obstructed duplicated ureter in an adult female: demonstration by magnetic resonance imaging.. Clin Imaging..

[10] Nzenza TC, Rice G, Kinnear N and Hennessey D (2016). An Interesting Case of Lifelong Urinary Incontinence.. Austin J Urol..

